# The responses of soil bacterial communities and enzyme activities to the edaphic properties of coal mining areas in Central China

**DOI:** 10.1371/journal.pone.0231198

**Published:** 2020-04-28

**Authors:** Jinhua Sun, Liu Yang, Jie Wei, Jine Quan, Xitian Yang

**Affiliations:** 1 College of Forestry, Henan Agricultural University, Zhengzhou, China; 2 Henan Ecological and Environmental Monitoring Center, Zhengzhou, China; University of Minnesota Twin Cities, UNITED STATES

## Abstract

Soil physicochemical properties, bacterial communities and enzyme activities change with land subsidence resulting from coal mining. However, research on the responses of bacterial communities and enzyme activities to the soil properties in different degree of subsidence areas is limited. As such, we collected soil samples from a control area (C area), a moderate mining subsidence area (M area) and a severe mining subsidence area (S area) in Central China. Soil properties, such as the pH, total nitrogen (TN) content, total phosphorus (TP) content, available phosphorus (AP) content, organic matter (OM) content, and soil enzyme (urease, invertase, catalase and alkaline phosphatase) activities were measured in each sampling area at depths of 0–20 cm, 20–40 cm, and 40–60 cm. The results indicated that the soil physiochemical properties, soil urease activity, soil alkaline phosphatase activity and soil bacterial richness and diversity in the topsoil (0–20 cm) of the mining subsidence area were significantly lower than those in the C area. However, the soil enzyme activities within the deepest layer of the subsidence area were significantly greater than those of the C area. The bacterial communities within the depth of 0–20 cm were dominated by *RB41*, *Pseudomonas*, *MND1*, *Nitrospira*, *Trichococcus*, *Sphingomonas* and *Dongia*, whereas *RB41* and *Pseudomonas* were the dominant species in the C area and subsidence area, respectively. Using correlation analysis, we found that the soil pH value, soil AP content and activities of the four enzymes were the main factors affecting the soil bacterial community structure. In addition, the soil nutrient contents, enzyme activities and bacterial richness and evenness decreased with increasing subsidence degree (classified by geological hazards, groundwater and landscape damage degree of coal mining subsidence). These results provide a reliable basis for environmental management of mining areas.

## 1. Introduction

Large-scale exploitation of coal resources has promoted economic development in China. However, this process has inevitably led to serious environmental problems [1]. More than 95% of coal mining occurs underground in China because the coal is buried at great depths. As a result, many ecological problems, such as land subsidence and cracking have occurred. Moreover, the soil hydraulic properties [2], soil nutrient distribution [3], and biological community [4] have been inevitably altered. Shallow hills surround the Pingdingshan mining area, and the ecological system is prone to severe soil erosion. Land subsidence aggravates soil erosion, decreases soil fertility, and leads to ecological degradation, and soil nutrients can infiltrate into deep layers in mining subsidence areas [4]. However, the vertical distribution of soil physiochemical properties in land subsidence areas has not been studied recently. As dominant regulators of soil OM decomposition and nutrient cycling, bacteria and enzymes play important roles in soil ecosystems. Enzyme activity can be used as an indicator of microbial activity [5] and plays a vital role in the decomposition of organic compounds, resulting in nutrient increases on the Loess Plateau [6]. Though the soil bacterial community and enzyme activity are sensitive to environmental change, their responses to soil properties in mining subsidence areas have not yet been studied.

The process of coal mining can alter the soil environment, such as the soil density, moisture [2] and nutrient distribution, all of which are closely related to bacterial diversity and enzyme activity. Soil enzymes are mainly derived from the activities of soil plants, animals and microbes. They are involved in various biochemical processes of soil, such as decomposition and synthesis of humus, decomposition of plant and microbial residues, hydrolysis and transformation of synthetic organic compounds, oxidation and reduction of inorganic compounds. Enzyme activities in collapsed ground were found to be lower than those of control groups whose ground was intact [7]. [8] found that the bacterial abundance was greater, and the bacterial community structure more complex in reclamation sites than in coal-excavated subsidence sites in mining fields in East China. [4] investigated the effect of post mining subsidence on soil bacterial diversity and its response to edaphic variables in the western China aeolian sand area. They found that soil microbial richness and diversity decreased due to land subsidence, and the main factors driving the changes in the bacterial community structure were the soil electronic conductivity, soil water content and soil depth. However, research rarely has been conducted on the effects of land subsidence on soil bacterial diversity and enzyme activity in hilly areas. In this study, we investigated the effects of different levels of mining subsidence on soil bacterial diversity and enzyme activities and how soil chemical properties responded in a hilly area.

## 2. Materials and methods

### 2.1 Sampling site description

**Fig 1:**
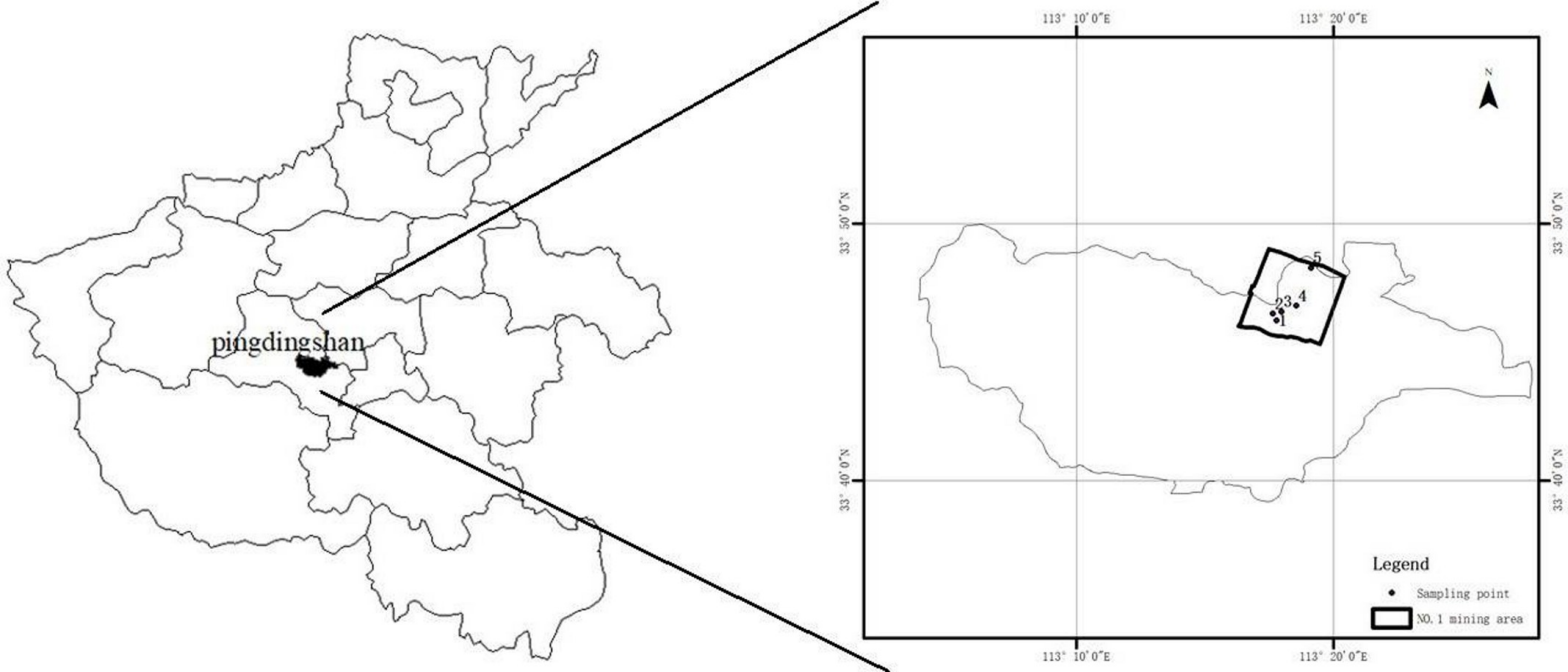
The descriptions of soil sample sites. Sample 1 and 2 were M areas; sample 3 and 4 were S areas, sample 5 was C area.

This study was conducted in the No.1mine, Pingdingshan city, Henan Province, Central China (113°11′45″-113°22′30″ E, 33°40′15″-33°48′45″ N) (Fig 1). These areas were investigated under the implementation of “Mine Geological Environment Protection and Rehabilitation Program” from Tian An coal mine of Pingdingshan. This region is located between Pingding and Luoniao Mountains, where the altitude ranges from 150 to 300 meters. The soil is mainly albic yellow cinnamon soil according to the soil classification system of the Food and Agriculture Organization-United Nations Educational, Scientific and Cultural Organization [9]; the soil erodes easily and contained inadequate nutrients to support vegetation. This region has a semiarid continental monsoon climate. The mean annual temperature is 14.9°C, the mean annual precipitation is 742.6 mm, the main period of rainfall occurs from July to September, and the mean annual evaporation is 2,157.8 mm. This study area was divided into three categories according to different subsidence degrees: S area, M area and C area (no subsidence) (Fig 1).

#### Permits obtained for the work

The mining area is located in the suburb of the city, it has been abandoned and left unattended. Therefore, no permits were required for sampling.

#### Ethics statement

The study was approved by Pingdingshan Coal Industry No.1 mine. The field studies did not involve endangered or protected species.

### 2.2 Sample collection

The subsidence area was mined in December 2011. Seven years after subsidence (June 2018), soil samples from 0–20 cm, 20–40 cm and 40–60 cm depths were collected from the S (S1, S2, and S3, respectively) area, M (M1, M2, and M3, respectively) area and C (C1, C2, and C3, respectively) area. Before sampling, three sampling plots (2 m×2 m) were established at each site. In each plot, three composite samples as replicates were randomly collected at each soil depth. After roots and stones were carefully removed, each 500 g soil sample was divided into three parts: one was air dried for determining the soil chemical properties, one was stored at -80°C for 16S rRNA gene analysis, and the rest was stored at 4°C for measuring the soil enzyme activity.

### 2.3 Soil analyses

#### 2.3.1 Physiochemical analysis

Soil property analysis was performed using standard soil test procedures. The soil pH values were measured at 1:2.5 soil/water ratios (w/v) via a pH meter [10]. The soil TN concentrations were determined by further grinding a subsample to particles 0.15 mm in size and igniting them in a high-induction N analyzer furnace (Elementar Americas Inc., Mt. Laurel, NJ). The TP of the soil was digested with perchloric acid and then measured by colorimetry. The soil OM was analyzed by volumetric determination with potassium dichromate, and the soil AP concentrations were measured via the molybdenum antimony colorimetric method [11].

#### 2.3.2 Enzyme activity

The soil catalase activity was measured with the methods of [12], while the soil alkaline phosphatase activity was measured according to the p-nitrophenyl phosphate method, as described by [13]. The soil urease activity was assayed on the basis of the amount of ammonia released: after compost samples and urea were incubated together; 1 N KCl and 0.01 N HCl were mixed to extract the ammonium, which was detected via the colorimetric method [14]. The soil invertase activity was estimated by the 3, 5-dinitrosalicylic acid (DNSA) method with sucrose used as a substrate.

#### 2.3.3 DNA extraction

The total genomic DNA was extracted from the samples using a Magen Hipure Soil DNA Kit (Meiji, China). The DNA concentration was monitored via a Qubit 3.0 fluorometer, and 20–30 ng of DNA was used for PCR and sequencing.

#### 2.3.4 16S rRNA gene amplification and purification

The V3-V4 regions of the 16S rRNA gene from each extract were amplified by PCR as follows: 2 min at 95°C; 25 cycles of 30 s at 95°C for denaturation, 30 s at 55°C for annealing and 45 s at 72°C for extension; and a final extension at 72°C for 10 min. A set of proprietary primers was designed to select relatively conserved regions bordering the V3 -V4 hypervariable regions of bacteria and Archaea16S rDNA. The V3 -V4 regions were amplified via forward primers containing the sequence “CCTACGGRRBGCASCAGKVRVGAAT” and reverse primers containing the sequence “GGACTACNVGGGTWTCTAATCC”. Moreover, indexed adapters were added to the ends of the 16S rDNA amplicons to generate indexed libraries ready for subsequent next-generation sequencing (NGS) on an Illumina MiSeq platform. PCR was performed in triplicate in a 25 μL mixture consisting of 2.5 μL of Trans Start Buffer, 2 μL of dNTPs, 1μL of each primer, and 20 ng of template DNA.

#### 2.3.5 Illumina MiSeq sequencing

DNA library concentrations were quantified via a Qubit3.0 fluorometer. To dilute the library to 10 nM, the DNA libraries were multiplexed and loaded on an Illumina MiSeq instrument according to the manufacturer’s instructions (Illumina, San Diego, CA, USA). The sequencing was performed on the PE250 platform; the image analysis and base calling were conducted with the MiSeq Control Software (MCS) program of the MiSeq instrument.

### 2.4 Statistical analysis

The QIIME 1.9.1 data analysis package was used for the 16S rRNA gene analysis. The forward and reverse reads were joined and assigned to samples on basis of their barcodes and were truncated by removing the barcode and primer sequences. Quality filtering of the joined sequences was performed, and sequences that did not fulfill the following criteria were discarded: sequence length <200 bp, no ambiguous bases, and mean quality score > = 20. The sequences were then compared with a reference database (RDP Gold database). The UCHIME algorithm was used to detect chimeric sequences, which were subsequently removed. The effective sequences were used in the final analysis. The sequences were grouped into operational taxonomic units (OTUs) by the clustering program VSEARCH (1.9.6) against the Silva 132 database that was preclustered at a 97% sequence identity. The ribosomal database program (RDP) classifier was used to assign taxonomic categories to all OTUs at a confidence threshold of 0.8. The RDP classifier uses the Silva 132 database which has taxonomic categories predicted at the species level. The sequences were flatten prior to the alpha and beta diversity statistical calculations. That is, we will take the same number of sequence from each sample and then analyze it later. The alpha diversity indices were calculated by QIIME from soil samples, the Shannon index was used to determine the bacteria diversity and the Chao1 index was used to determine the richness. Weighted and unweighted UniFrac distances were calculated to estimate beta diversity with non-metric multidimensional scale (NMDS). An unweighted pair group method with arithmetic mean (UPGMA) tree constructed on the basis of the beta diversity distance matrix.

Correlations between the soil properties and bacterial communities were determined by SPSS 22.0. Canonical correspondence analysis (CCA) was used to analyze the relationships between the bacterial community and environmental factors in R 3.5.1 in conjunction with chi-square test. Differences between the samples were calculated via one-way analysis of variance (ANOVA) in conjunction with a p test at the level of 0.05.

## 3. Results

### 3.1 Soil enzyme activities

**Fig 2:**
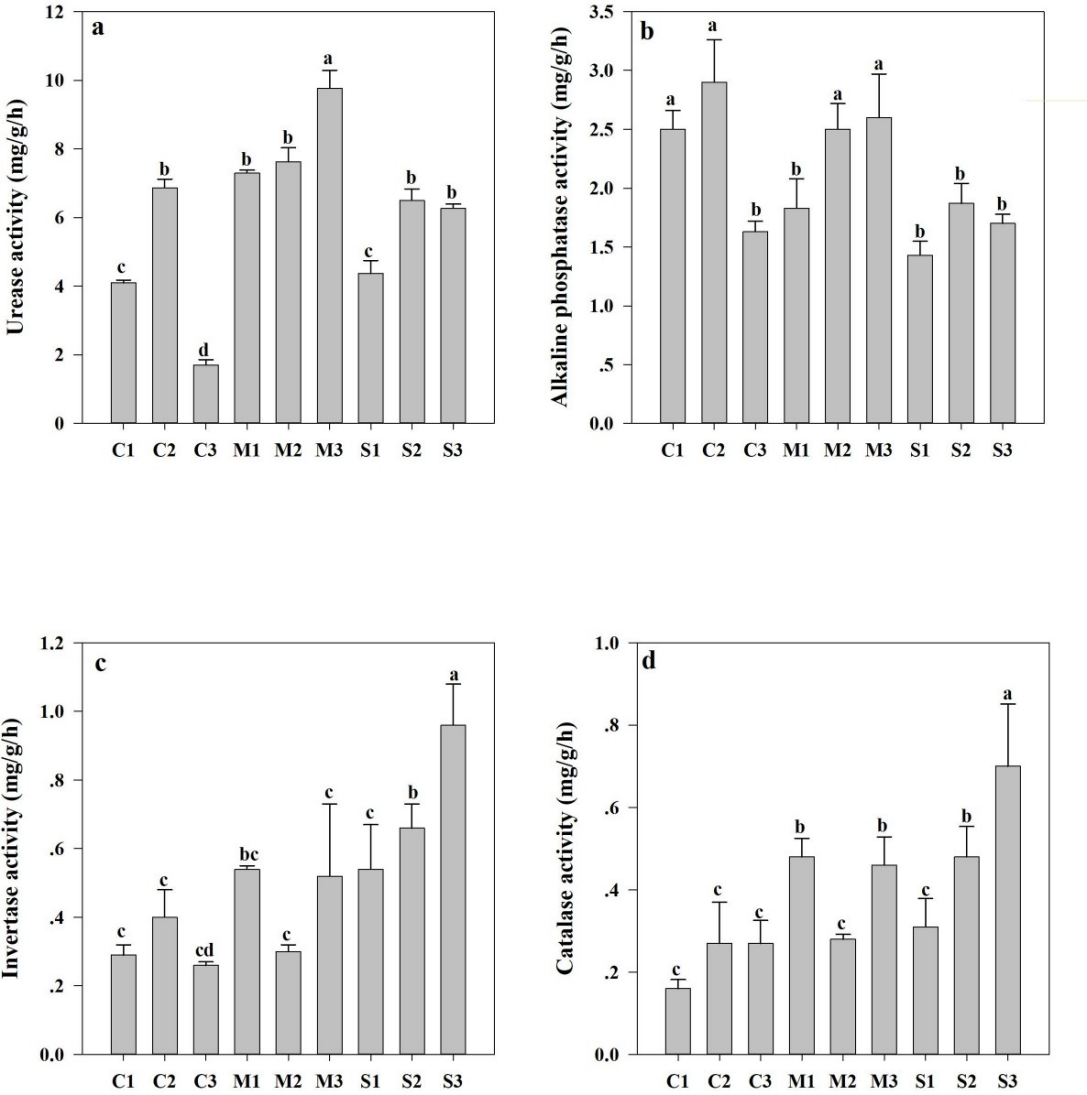
Enzyme activities in soils subjected to different degrees of land subsidence. The data are expressed as the mean ± the SE of three biological replicates per treatment. The values in each panel that are labeled with different letters are significantly different at p<0.05. C1, 0–20 cm depth in the control area; C2, 20–40 cm depth in the control area; C3, 40–60 cm depth in the control area; M1, 0–20 cm depth in the moderate subsidence area; M2, 20–40 cm depth in the moderate subsidence area; M3, 40–60 cm depth in the moderate subsidence area; S1, 0–20 cm depth in the severe subsidence area; S2, 20–40 cm depth in the severe subsidence area; S3, 40–60 cm depth in the severe subsidence area.

The soil enzyme activities varied with the degree of land subsidence. The soil urease activity was greatest in the M area at a depth of 40–60 cm (Fig 2A); as the degree of subsidence increased, the soil alkaline phosphatase activity decreased. The soil alkaline phosphatase activity at a depth of 0–40 cm was significantly greater than that at a depth of 40–60 cm in the C area. The opposite results were recorded in the M and S areas (Fig 2B). The soil invertase activity at a depth of 40–60 cm was significantly greater than that at a depth of 0–20 cm in both the M and S areas (Fig 2C). Moreover, the soil catalase activity was great throughout the topsoil in three areas, and increased with increased subsidence degree (Fig 2D).

### 3.2 Soil physiochemical properties

The three analyzed groups differed with respect to the soil nutrient indicators, such as the soil OM, TN, TP and AP contents and pH value (Table 1). The soil TP and TN contents at the same depth in the C area were generally greater than those in the M and S areas; In addition, the contents were significantly greater at the 0–20 cm than at other depths in all three areas. The soil pH at the same depth was significantly greater in the C area than in the M and S areas. Moreover, the pH at 0–20 cm was significantly greater than those at 20–60 cm in the M and S areas. The soil OM content decreased significantly with increasing subsidence. However, the differences were not significant among all soil layers, indicating that the vertical distribution of OM in the soil was relatively stable. In the C area, the soil AP content was significantly highest at 40–60 cm, while the opposite occurred in the M and S areas.

**Table 1:** Soil physiochemical properties in the coal mining areas.

	C1	C2	C3	M1	M2	M3	S1	S2	S3
pH	8.20±0.02b	8.36±0.02a	8.20±0.08b	7.60±0.08d	8.01±0.02c	5.39±0.02g	5.95±0.01f	6.96±0.02e	6.90±0.01e
AP(mg/kg)	4.32±0.01i	4.79±0.01f	6.65±0.01b	6.3±0.08c	4.69±0.01g	5.25±0.01d	11.09±0.02a	5.12±0.01e	4.49±0.08h
TP(g/kg)	0.30±0.08a	0.34±0.01a	0.26±0.01a	0.22±0.02b	0.22±0.01b	0.23±0.01b	0.11±0.01c	0.04±0.01d	0.05±0.01cd
OM(g/kg)	9.1±0.23a	8.5±0.12b	7.5±0.17c	8.0±0.23b	6.4±0.06c	5.8±0.23d	5.7±0.12d	4.2±0.29e	3.1±0.12f
TN(g/kg)	1.30±0.08a	0.58±0.08b	0.59±0.16b	0.71±0.08b	0.51±0.16c	0.49±0.08c	0.41±0.08c	0.8±0.08b	0.39±0.08c

AP, available P; TP, total P; OM, organic matter; TN, total N; TC, total C. The different lowercase letters indicate significant differences between the different soil samples. C1, 0–20 cm depth in the control area; C2, 20–40 cm depth in the control area; C3, 40–60 cm depth in the control area; M1, 0–20 cm depth in the moderate subsidence area; M2, 20–40 cm depth in the moderate subsidence area; M3, 40–60 cm depth in the moderate subsidence area; S1, 0–20 cm depth in the severe subsidence area; S2, 20–40 cm depth in the severe subsidence area; S3, 40–60 cm depth in the severe subsidence area. On each line, the means ± SDs followed by different lowercase letters represent significantly different at p<0.05.

### 3.3 Soil bacterial community composition

**Fig 3:**
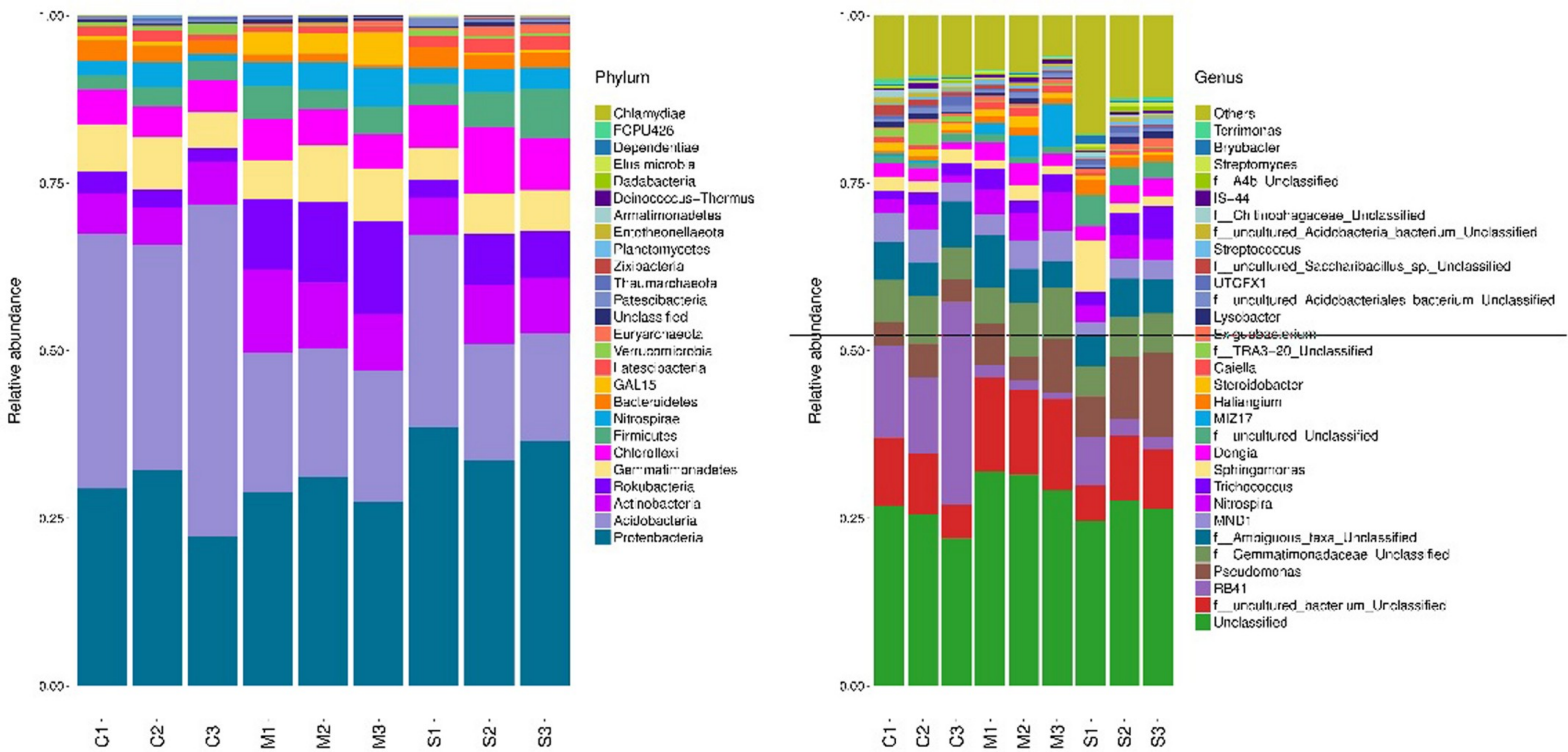
Bacterial community compositions relative abundance at the phylum (a) and genus (b) levels of 9 samples.

*Proteobacteria* was the dominant phylum in all samples in the different areas (22.25%–38.68%), followed by *Acidobacteria* (16.07%–49.57%), *Actinobacteria* (5.5%–12.31%) (Fig 3A) and *Rokubacteria* (2.1%–13.83%). These phyla accounted for more than 70% of the bacteria within the soil samples. *Proteobacteria* was the most abundant phylum at the 20–40 cm depth in each sample. The relative abundance of *Acidobacteria* was greatest in the deep layer (40–60 cm) in the C area, and lowest in the topsoil (0–20 cm) in the M and S areas, indicating that coal mining subsidence altered the distribution of the bacterial species in the soil in the vertical direction. The differences in the relative abundance of the *Actinobacteria* phylum among all soil samples were not significant. In comparison, the relative abundance of the *Rokubacteria* phylum was greater in the M area than that in the C and S areas.

The seven most abundant genera that have been classified included *RB41* (1.01%–30.28%), *Pseudomonas* (3.36%–12.54%), *MND1* (1.96%–4.88%), *Nitrospira* (0.98%–5.8%), *Trichococcus* (1.24%–4.85%), *Sphingomonas* (1.16%–7.72%) and *Dongia* (1.08%–3.21%) (Fig 3B). The relative abundance of *RB41* within the same layer was significantly greater in the C area than in the M and S areas, while that of *Pseudomonas* was the opposite. There were no significant differences in the relative abundance of *MND1* among any of the samples from the three areas. The relative abundances of *Nitrospira*, *Trichococcus* and *Dongia* were greatest in the topsoil (0–20 cm) in the C area, and greatest in the deepest layer (40–60 cm) in the S area. However, there were no significant differences in the relative abundances of *Nitrospira*, *Trichococcus* or *Dongia* among the three layers in the M area. The relative abundance of *Sphingomonas* was significantly greater in the topsoil (0–20 cm) in the S area than in the other layers and in the other areas (Fig 3B).

### 3.4 Soil bacterial richness and diversity

A total of 401,972 high-quality reads were obtained from the soils in the C, M and S areas after 509,567 raw reads were filtered (ranging from 46,865 to 72,159) via the clustering program Vsearch 1.9.6, which resulted in 1,294, 1,284, and 1,296 OTUs, respectively, the clustering threshold is 97%. The sample numbers satisfied the OTU determination based on rarefaction curves (S1 Fig). The greatest relative abundance of OTUs was approximately 10%, and the diversity and evenness of the OTUs in the S area were greater than those in the C area (S2 Fig). A Venn diagram was constructed showing that the three areas shared 426 OTUs (S3 Fig), and more than 90% of the OTUs from all soil samples belonged to the shared OTUs. Land subsidence influenced the bacterial richness and diversity indices, which ranged 683–767 (Ace richness index), 690–792 (Chao richness index), 6.80–7.90 (Shannon diversity index), and 0.95–0.99 (Simpson diversity index) (Table 2). These four indices changed similarly with depth. However, in the S area, the Ace and Chao indices in the deepest layer (40–60 cm) were greater than those in the other layers. The values of the Ace, Chao, Shannon and Simpson indices were greater in the S area than in the C area at the same depth. These results indicated that the bacterial richness and diversity decreased with depth in the M area, but increased with depth in the S area.

**Table 2:** The soil bacterial richness and diversity indices.

Sample code	OUT	Ace	Chao	Good Coverage	Shannon	Simpson
C1	434±11.55a	763±15.15a	769±18.16ac	0.99±0.00a	7.90±0.03a	0.99±0.00a
C2	430±12.12a	739±4.10ab	743±8.47bc	0.99±0.00c	7.80±0.05ab	0.99±0.00a
C3	430±5.20a	683±14.27b	690±16.27de	0.99±0.00ab	6.80±0.02d	0.95±0.00c
M1	426±6.35a	758±5.03a	769±8.87ab	0.99±0.00ab	7.61±0.02ab	0.99±0.00a
M2	429±12.12a	741±9.26a	748±12.13b	0.99±0.00a	7.70±0.01a	0.99±0.00a
M3	429±9.24a	710±8.33b	715±5.55e	0.99±0.00a	7.19±0.19c	0.98±0.01b
S1	438±11.55a	763±3.94a	788±8.71a	0.99±0.00ad	7.73±0.03a	0.99±0.00ab
S2	429±5.77a	741±3.98a	759±2.79ab	0.99±0.00cd	7.83±0.08ab	0.99±0.00ab
S3	429±8.66a	767±15.42a	792±13.94a	0.99±0.00a	7.64±0.15ab	0.98±0.01b

C1, 0–20 cm depth in the control area; C2, 20–40 cm depth in the control area; C3, 40–60 cm depth in the control area; M1, 0–20 cm depth in the moderate subsidence area; M2, 20–40 cm depth in the moderate subsidence area; M3, 40–60 cm depth in the moderate subsidence area; S1, 0–20 cm depth in the serious severe subsidence area; S2, 20–40 cm depth in the severe subsidence area; S3, 40–60 cm depth in the severe subsidence area. On each line, the means ± SDs followed by different lowercase letters are significantly different at p<0.05.

### 3.5 Soil microbial community structure

**Fig 4:**
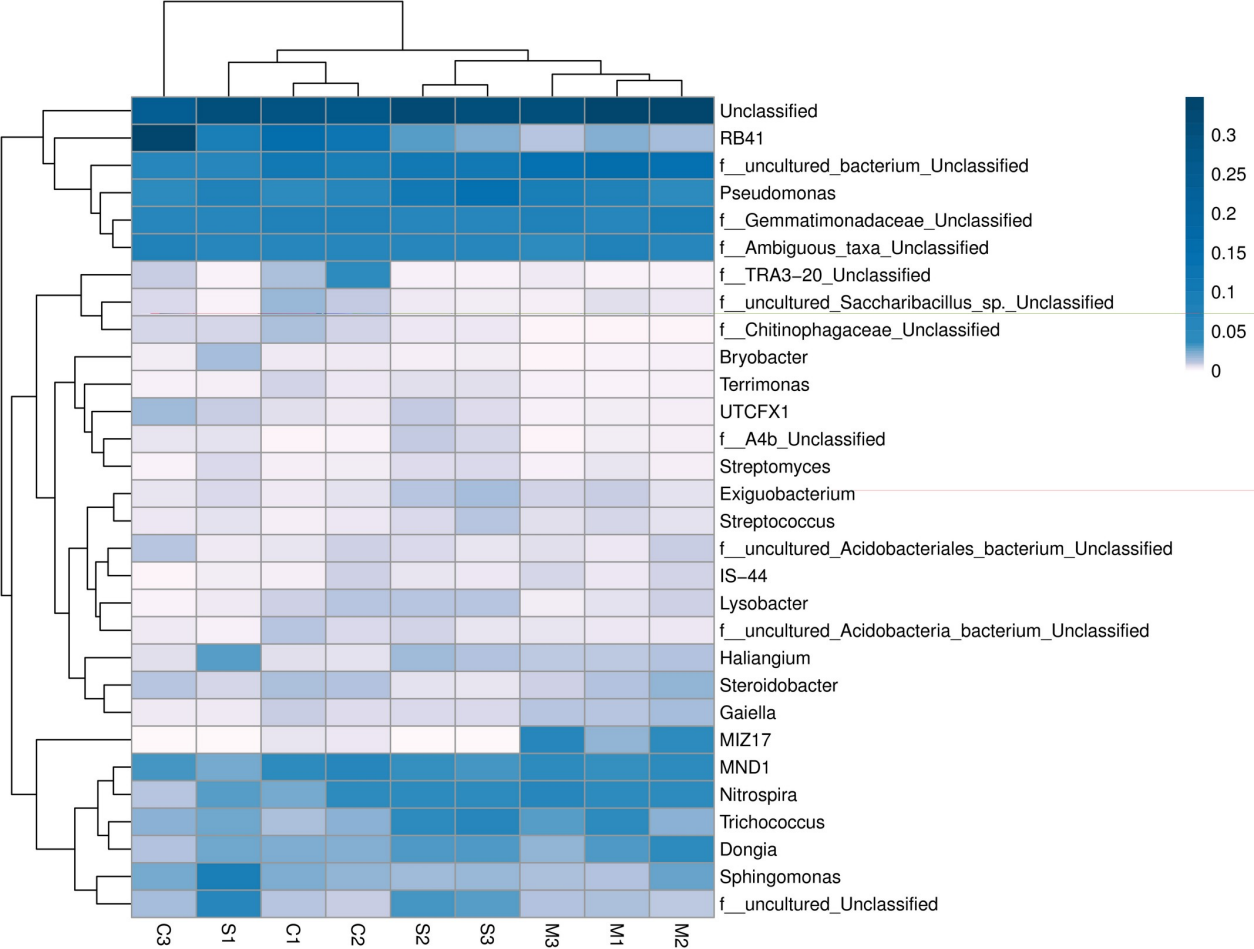
Heatmap showing the distribution of the 50 most abundant genera among the 9 samples. The relative percentage values of the microbial genera are indicated by different hues. The color blocks and color gradients represent the relative abundance values and the dissimilarity of the microbial community composition, respectively.

The NMDS analysis (stress value <0.033) was based on the beta diversity distance matrix calculated from the species composition among all the samples (S4 Fig). The results showed that the C1, C2 and C3, M1, M2 and M3, and S1, S2 and S3 sample were grouped close together, suggesting that the soil bacterial diversity was affected mainly by the degree of subsidence. The soil bacterial diversity in each of the C and M areas was more similar than that in the S area; moreover, the distance between the deepest layer (40–60 cm) and the other layers (0–40 cm) was greater in the C and M areas than in the S area. This finding indicated that severe subsidence disrupted the soil bacterial community. Moreover, the soil bacterial community in the topsoil (0–20 cm) in the S area was significantly changed by land subsidence. A heatmap was constructed to display the relative abundances and dissimilarity in the microbial community composition among the samples at the gene level (Fig 4). For instance, the relative abundance of *RB41* (30.28%) in the C3 area increased by 121.0 2%, 166.31% and 321.14% respectively and significantly compared with C1, C2 and S1 areas, whereas was less abundant in the all the depths of M area. In comparison, *MIZ17* was abundant in the M areas but deficient in other areas; the relative abundance of *Pseudomonas* in the S2 area (9.35%) was 4.59 times, 1.61times and 1.69 times higher than that of S3, C1 and C3 areas respectively. The relative abundance of *Trichococcus* and *Dongia* were more in the S area than others.

### 3.6 Correlations between bacterial communities and soil properties

Pearson’s correlation coefficients between soil properties and bacterial communities are shown in Table 3. *RB41* was positively correlated with the pH (p<0.05) and catalase activities (p<0.05) but negatively correlated with the urease and alkaline phosphatase activities (p<0.05). *Pseudomonas* was significantly negatively correlated with OM (p<0.01), TP contents (p<0.05) and the urease and alkaline phosphatase activities (p<0.05) but significantly positively correlated with the catalase activity (p<0.01) and invertase activity (p<0.05), and *MND1* was significantly positively correlated with AP and TP contents (p<0.05). *Nitrospira* was significantly negatively correlated with catalase activity (p<0.05) and invertase activity (p<0.05). *Trichococcus* was significantly negatively correlated with TP and OM contents (p<0.05) but significantly positively correlated with the catalase activity (p<0.01) and invertase activity (p<0.05). There was a significant positive correlation between *Sphingomonas* and AP content (p<0.01). *Dongia* was significantly negatively correlated with the catalase activity (p<0.05). All bacteria were significant correlated with the urease and alkaline phosphatase activities (p<0.05). Therefore, the soil chemical properties and enzyme activities in the study area had significant effects on the bacterial communities. In contrast, The TP, AP, and OM contents and the activities of four enzyme played vital roles in forming the structure of the bacterial communities.

**Table 3:** Pearson’s correlation coefficients between the relative abundance of the 7 dominant bacterial genera and soil properties.

	*RB41*	*Pseudomonas*	*MND1*	*Nitrospira*	*Trichococcus*	*Sphingomonas*	*Dongia*
pH	0.5*	-0.559*	0.317	-0.484	-0.395	-0.335	0.021
AP	0.139	-0.126	0.694*	-0.325	-0.163	0.889**	-0.233
TP	0.46	-0.768*	0.714*	-0.025	-0.755*	-0.222	-0.42
OM	0.482	-0.837**	0.498	-0.238	-0.793*	0.075	-0.338
TN	0.236	-0.351	0.334	-0.29	-0.392	-0.288	-0.073
CAT	0.715*	0.996**	-0.968*	-0.955*	0.999**	-0.540	-0.780*
US	-0.861*	-0.944*	0.995**	0.990**	-0.961*	0.726*	0.907*
AlP	-0.872*	-0.937*	0.992**	0.987*	-0.955*	0.740*	0.916*
INS	0.406	0.960*	-0.808*	-0.886*	0.943*	-0.191	-0.494

AP, available P; TP, total P; OM, organic matter; TN, total N; CAT, catalase activity; US, urease activity; AlP, alkaline phosphatase activity; INS, inverse activity. The different lowercase letters indicate significant differences between the soil samples. The correlations are significant at the 0.05 level.

* indicates p < 0.05;

** indicates p < 0.01.

**Fig 5:**
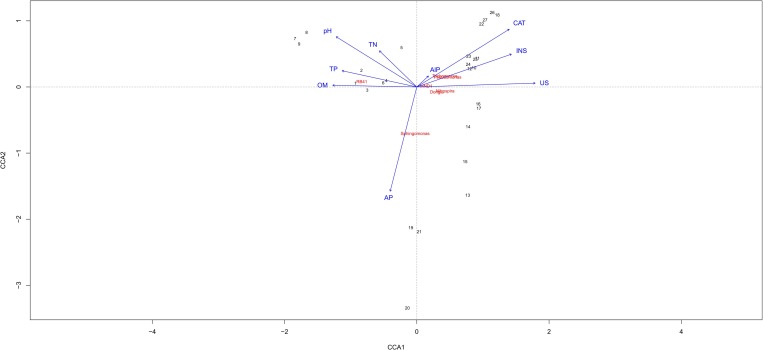
Canonical correspondence analysis (CCA) of the bacterial diversity and soil properties. TN, total N; AP, available P; OM, organic matter; TP, total P. CAT, catalase activity; US, urease activity; AlP, alkaline phosphatase activity; INS, inverse activity. The solid arrows represent the soil properties and enzyme activities, and different numbers represent samples from the control, moderate and severe subsidence areas.

The objective of the CCA was to identify the main environmental factors affecting the bacterial community structure (Fig 5). Among the soil chemical properties, the soil pH and AP and TP contents and the soil catalase and urease activities played vital roles in forming the bacterial community structure (S3 Table). *RB41* was significantly positively correlated with the soil pH and soil TN, TP and OM contents in the C area. Moreover, there was a significant correlation between *Sphingomonas* and the soil AP content in the M area. *Trichococcus*, *Pseudomonas* and *MND1* were significantly correlated with the activities of four enzyme in the S area (Fig 5). The variance results from the principal component analysis showed that the simulation model had a good degree of fit (p<0.001, S1 Table), and the significant eigenvalues of the variance analysis are shown in S2 Table. The bacterial communities were divided into four groups, which indicated that the degree of subsidence and soil depth affected the soil bacterial community structure.

## 4. Discussion

### 4.1 Effects of land subsidence on soil enzyme activities and physiochemical properties

In this study, with increase of soil depth, the soil invertase, urease and alkaline phosphatase activities decreased significantly (Fig 2). These findings were in accordance with previous results [7]. This phenomenon can be explained by the distribution of soil nutrients within different layers, for the enzyme reactions are based on nutrients [15]. Land subsidence decreased the soil urease and alkaline phosphatase activities but increased the soil catalase and invertase activities (Fig 2). Soil urease and alkaline activities are the main factors that regulate N and P cycling [16]. N and P losses caused by subsidence caused a decrease in enzyme activities [4]. Invertase catalyzes the hydrolysis of sucrose into glucose and fructose to provide carbon for plants and microbes. One possible explanation might be that the soil OM content in the C area was greater than S area, and soil microbes may synthesize additional catalase and invertase to adapt to unfavorable environments.

Land subsidence severely deformed the surface and redistributed the nutrients in the vertical direction of soil. In the present study, with increase of soil depth and subsidence extent, the soil OM, TN and TP contents decreased (Table 1). These results were consistent with those from the study of [3]. Land subsidence and cracking can alter the soil bulk density and porosity [17; 18]. With the precipitation soluble nutrients such as C, N and P are lost along many pores, which leads to the OM, TN and TP decrease in the subsidence areas. In addition, subsidence can aggravate soil erosion and OM composition [19]. In the present study, soil pH in the M and S areas increased with increase of soil depth. But the soil AP content decreased in the M and S areas and increased in the C area (Table 1). The soil AP content in the C area was affected mainly by the runoff infiltration. However, a large amount of AP was released onto the surface from the decomposition of phosphoric minerals in the acidic environment in the M and S area.

### 4.2 Variation in soil bacterial community composition by land subsidence

Bacterial community structure are affected by many ecological factors. For instance, climate conditions can affect the abundance of genes from *Actinobacteria*, *Planctomycetes* and *Proteobacteria* [20]. A sequencing study revealed that *Actinobacteria* can outcompete other dominant groups, such as *Acidobacteria* under arid conditions [21]. [22] reported that different plant species change the composition of soil bacterial community by affecting the soil pH. *Proteobacteria*, *Acidobacteria*, *Actinobacteria* and *Rokubacteria* were the dominant bacterial phyla in the present study, accounting for approximately 80–85% of the total OTUs. This result implies that these phyla may play important roles in coal mining areas. The climate and environmental conditions were identical among the sites in this study, but the subsidence levels differed; thus, the conditions were appropriate for analyzing how soil properties affecting soil bacterial diversity and bacterial communities.

The process of coal mining can alter the soil environment, through actions such as acidifying, compacting and eroding the soil, which directly affected the bacterial community. The results of the present study showed that the soil bacterial community composition varied among the three studied areas. The diversity and richness of the bacterial community decreased with increasing of subsidence level and soil depth. Though the numbers of bacterial communities were lowest in the S area, they play an important role in the regulation of biogeochemical cycles [23; 24; 25]. In addition, anthropogenic soil disturbances (such as agriculture, mining, and the disposal of residues or waste) shift ecosystem equilibria, forcing microorganisms to adapt to new habitats [26]. In the present study, the dominant bacterial communities comprised *RB41*, *Pseudomonas*, *MND1*, *Nitrospira*, *Trichococcus*, *Sphingomonas* and *Dongia* in the C and S areas. *RB41*, belongs to *Acidobacteria* and exhibited a relatively range of tolerance to pH (up to 10). Some studies have reported that *RB41* might play an important role in maintaining the metabolic and biogeochemical function of soil under long-term low-nutrient stress conditions. Our results were the opposite; one explanation is that the pH was greater in the C area than that in the S area, and the conditions were more suitable for *RB41* to survive in the C area. However, *Pseudomonas* was the most abundant bacteria in the S area, and members of this genus have demonstrated a great deal of metabolic diversity. The higher abundance of *Pseudomonas* is linked with soil health [27; 28].The organisms in the *Pseudomonas* genus are mostly free-living bacteria that are widely distributed among soil, water, and marine environments and are present on the skin of animals, including humans. They are strict aerobes and move via one or more polar flagella. Land subsidence results in the creation of many soil pores, satisfying the bacterial demand for oxygen.

### 4.3 Response of soil bacterial community and enzyme activities to soil properties

In the present study, the effect of soil urease, catalase, and invertase activities on the bacterial community structure was greater than that of alkaline phosphatase activity (Fig 5). Soil enzyme activities are the main way for microbes to decompose nutrients and OM [29] and are relatively more representative of bacterial physiology [30; 31].

Physiochemical soil parameters are the major influencing factors for soil microbial diversity [32; 33]. We found that various physicochemical soil factors had different effects on the bacterial diversity in areas affected by coal mine subsidence; specifically, soil pH and AP content were most significant in shaping soil bacterial communities. Many studies have demonstrated that pH is a major factor determining soil bacterial community composition [34; 20]. pH was a key factor affecting the diversity, structure, interaction, and function of rhizosphere bacterial communities in crop soil under control conditions [35]. The soil pH affects the protein synthesis of *Acidobacteria*, *Actinobacteria* and *Cyanobacteria* [20]. In addition, *Acidobacteria* had the function of degrading plant reidue multimers [36; 37], which provided nutrient input to those that survive under acidic conditions. Some study have also found that soil pH was correlated with soil bacterial phylogenetic diversity at the continental scale, with peak diversity in soil with near-neutral pH [38]. That may be the major reasons why soil pH was closely correlated with soil bacterial communities.

In addition, the soil AP content was positively correlated with the abundance of *MND1* and *Sphingomonas* (belong to *Proteobacteria*). The growth rate hypothesis emphasizes that the growth of most bacteria is related to the need for P for ribosomal RNA synthesis, prompting the bacterial community to enter the soil [39]. Bacteria play important roles in the mineralization of organic P and dissolution of inorganic P. Microbial inorganic P solution occurs primarily through release of organic anions, which causes precipitated P-metal complexes to solubilize, releasing inorganic P [40; 41]. Bacteria capable of this mode of solution include members of *Pseudomonadaceae*, *Rhizobiaceae*, *Caulobacteraceau*, *Comamonadaceae*, *Burkholderiaceae*, and *Enterobacteriaceae* within *Proteobacteria* [42].

This study found land subsidence influenced soil bacterial community richness and diversity, these effects were realized by changes of soil physiochemical properties and enzyme activities. However, the correlations between the soil properties and the microbial communities differ among soil systems. For instance, [43] reported a significant correlation between soil *Actinobacteria* and *Proteobacteria* and soil OM, TN and TP. [10] observed the effects of soil properties on the bacterial communities present in soil from the Shengli Oilfield and found that soil TP was positively correlated with *Actinobacteria* but negatively correlated with *Firmicutes*.

## 5. Conclusion

Mining-induced subsidence significantly decreased the soil enzyme activities in the topsoil, especially those of soil invertase, urease and alkaline phosphatase, In addition, the soil pH, and soil TN, TP and OM contents decreased in response to subsidence, and the soil properties and bacterial community diversity and richness at a depth of 0–20 cm were more susceptible to coal mining. The dominant bacterial communities comprised *RB41*, *Pseudomonas*, *MND1*, *Nitrospira*, *Trichococcus*, *Sphingomonas* and *Dongia*. The main environmental factors determining the soil bacterial community in the coal mining areas of Central China were the pH and AP content, which determine the protein and RNA synthesis of bacteria. In short, the soil nutrients, enzyme activities and soil microbial community structure were disturbed by land subsidence. Therefore, it is necessary to take measures to improve the environment of subsidence areas.

## Supporting information

S1 Fig Rarefaction curves of observed OTUs. C1, 0–20 cm depth in the control area; C2, 20–40 cm depth in the control area; C3, 40–60 cm depth in the control area; M1, 0–20 cm depth in the moderate subsidence area; M2, 20–40 cm depth in the moderate subsidence area; M3, 40–60 cm depth in the moderate subsidence area; S1, 0–20 cm depth in the severe subsidence area; S2, 20–40 cm depth in the severe subsidence area; S3, 40–60 cm depth in the severe subsidence area.
(TIF)

S2 Fig The OUT rank curve of soil samples of three areas. C1, 0–20 cm depth in the control area; C2, 20–40 cm depth in the control area; C3, 40–60 cm depth in the control area; M1, 0–20 cm depth in the moderate subsidence area; M2, 20–40 cm depth in the moderate subsidence area; M3, 40–60 cm depth in the moderate subsidence area; S1, 0–20 cm depth in the severe subsidence area; S2, 20–40 cm depth in the severe subsidence area; S3, 40–60 cm depth in the severe subsidence area.
(TIF)

S3 Fig Venn diagram of nine soil samples of three areas. C1, 0–20 cm depth in the control area; C2, 20–40 cm depth in the control area; C3, 40–60 cm depth in the control area; M1, 0–20 cm depth in the moderate subsidence area; M2, 20–40 cm depth in the moderate subsidence area; M3, 40–60 cm depth in the moderate subsidence area; S1, 0–20 cm depth in the severe subsidence area; S2, 20–40 cm depth in the severe subsidence area; S3, 40–60 cm depth in the severe subsidence area.
(TIF)

S4 Fig NMDS diagram of all soil samples of three areas. C1, 0–20 cm depth in the control area; C2, 20–40 cm depth in the control area; C3, 40–60 cm depth in the control area; M1, 0–20 cm depth in the moderate subsidence area; M2, 20–40 cm depth in the moderate subsidence area; M3, 40–60 cm depth in the moderate subsidence area; S1, 0–20 cm depth in the severe subsidence area; S2, 20–40 cm depth in the severe subsidence area; S3, 40–60 cm depth in the severe subsidence area.
(TIF)

S1 Table Variance for principal component analysis. (DOCX)

S2 Table Significant variance analysis of eigenvalues. (DOCX)

S3 Table Coefficient variance analysis of principal components of environmental factors. (DOCX)
