# Supplementary figures and images for: The responses of soil bacterial communities and enzyme activities to the edaphic properties of coal mining areas in Central China
Source: PLoS One. 2020 Apr 28;15(4):e0231198. doi: 10.1371/journal.pone.0231198 (PMC7188301; doi:10.1371/journal.pone.0231198)

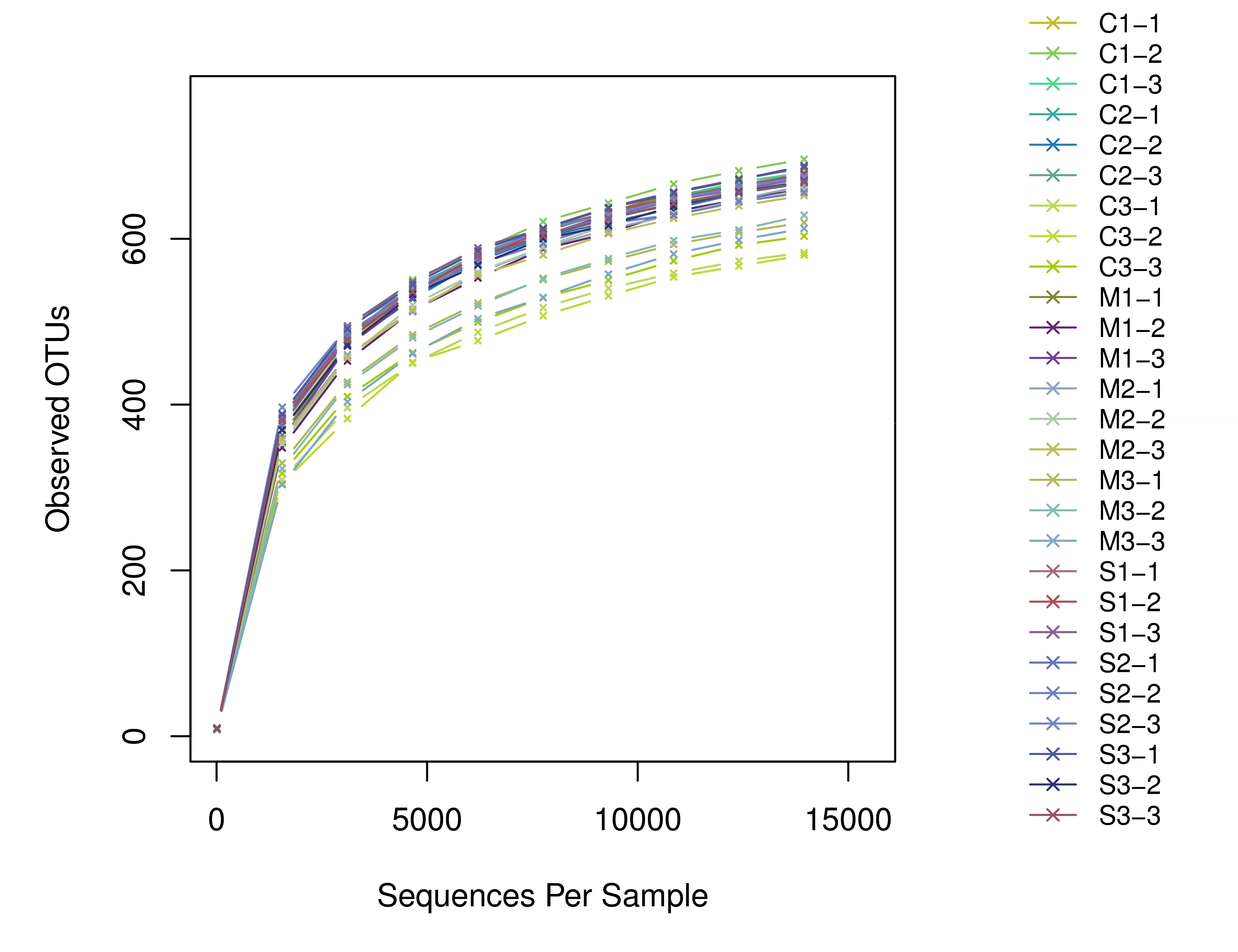

Supplement: S1 Fig — C1, 0–20 cm depth in the control area; C2, 20–40 cm depth in the control area; C3, 40–60 cm depth in the control area; M1, 0–20 cm depth in the moderate subsidence area; M2, 20–40 cm depth in the moderate subsidence area; M3, 40–60 cm depth in the moderate subsidence area; S1, 0–20 cm depth in the severe subsidence area; S2, 20–40 cm depth in the severe subsidence area; S3, 40–60 cm depth in the severe subsidence area. (TIF) [file pone.0231198.s001.tif]

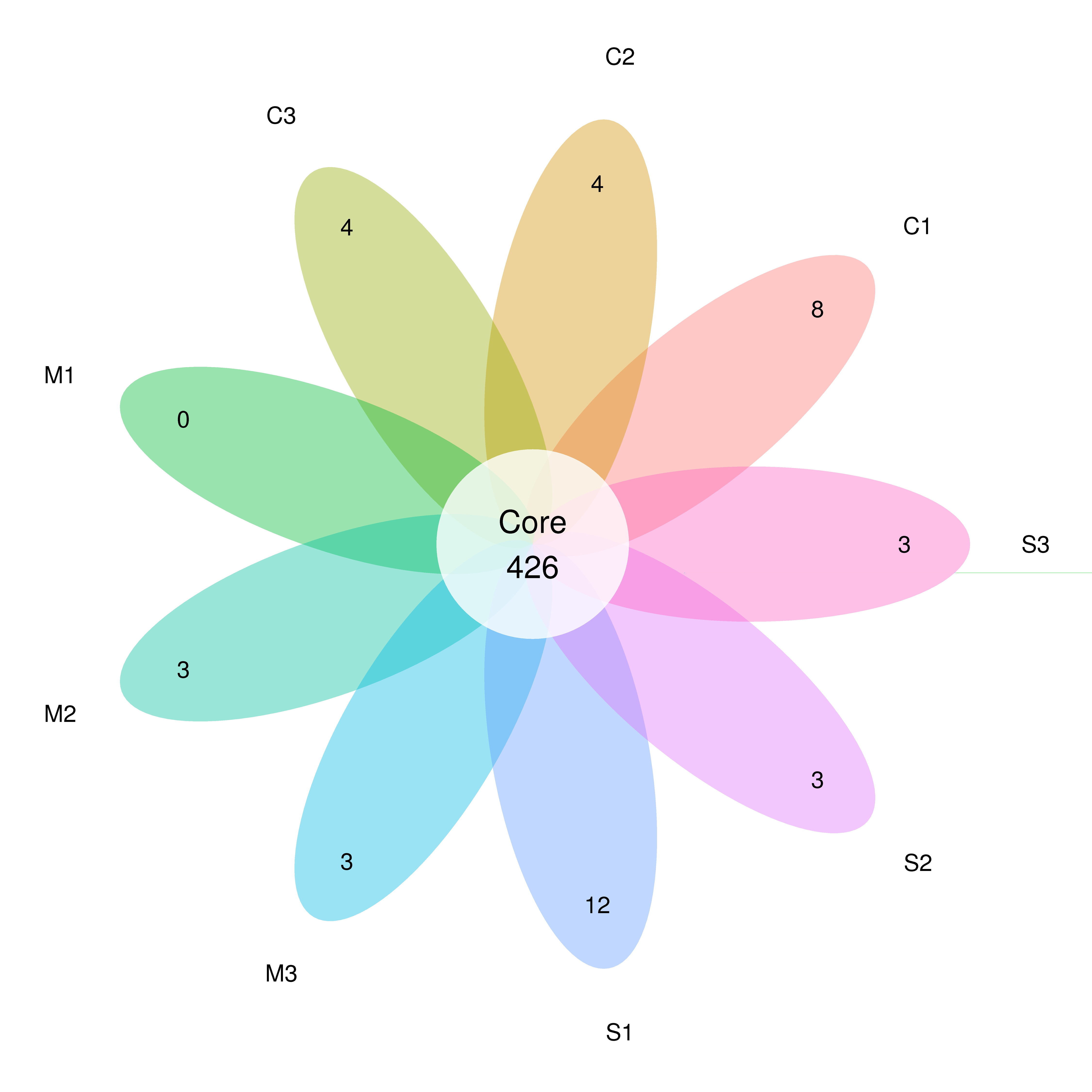

Supplement: S3 Fig — C1, 0–20 cm depth in the control area; C2, 20–40 cm depth in the control area; C3, 40–60 cm depth in the control area; M1, 0–20 cm depth in the moderate subsidence area; M2, 20–40 cm depth in the moderate subsidence area; M3, 40–60 cm depth in the moderate subsidence area; S1, 0–20 cm depth in the severe subsidence area; S2, 20–40 cm depth in the severe subsidence area; S3, 40–60 cm depth in the severe subsidence area. (TIF) [file pone.0231198.s003.tif]

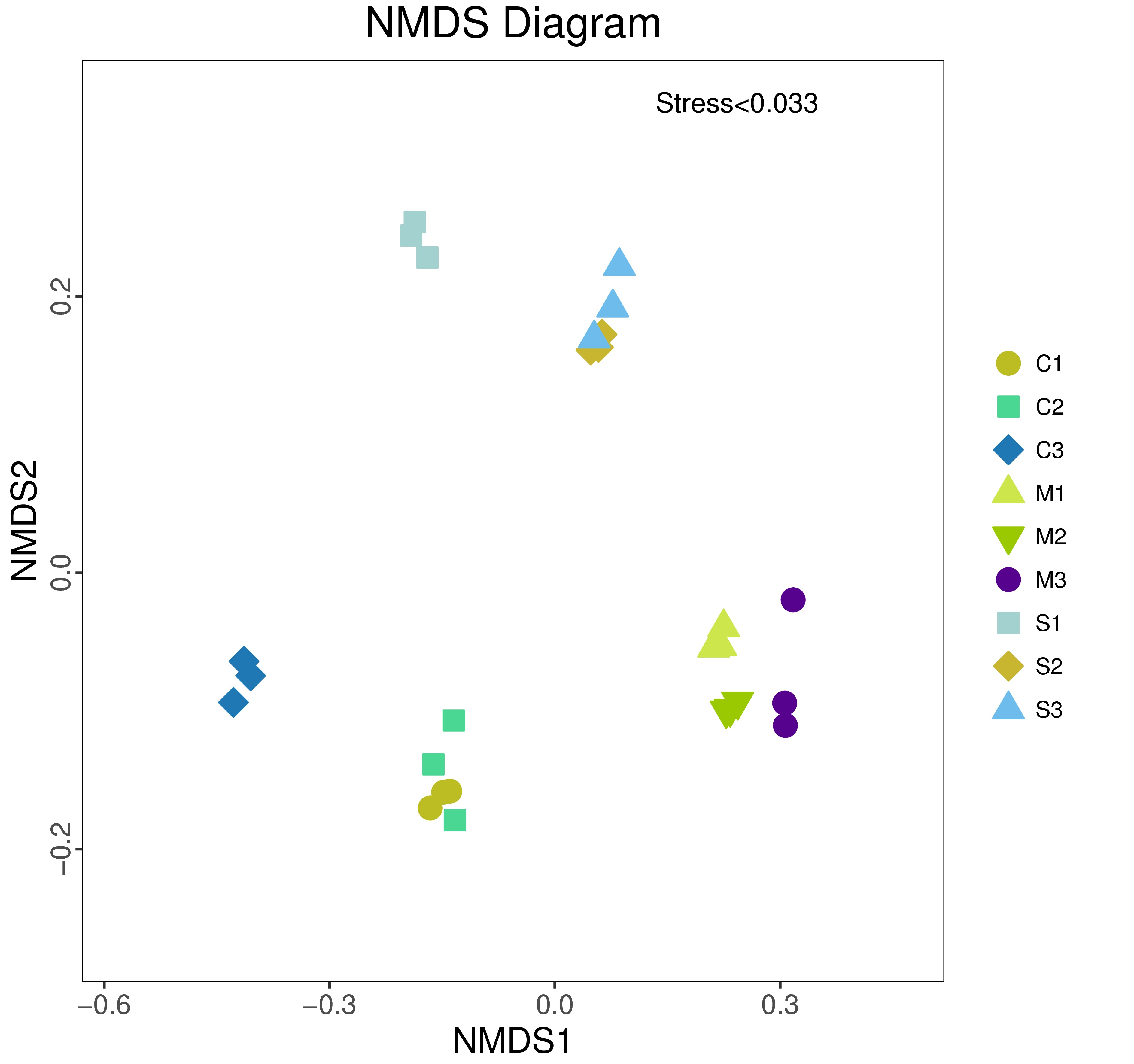

Supplement: S4 Fig — C1, 0–20 cm depth in the control area; C2, 20–40 cm depth in the control area; C3, 40–60 cm depth in the control area; M1, 0–20 cm depth in the moderate subsidence area; M2, 20–40 cm depth in the moderate subsidence area; M3, 40–60 cm depth in the moderate subsidence area; S1, 0–20 cm depth in the severe subsidence area; S2, 20–40 cm depth in the severe subsidence area; S3, 40–60 cm depth in the severe subsidence area. (TIF) [file pone.0231198.s004.tif]
