# Supplementary material for: The responses of soil bacterial communities and enzyme activities to the edaphic properties of coal mining areas in Central China
Source: PLoS One. 2020 Apr 28;15(4):e0231198. doi: 10.1371/journal.pone.0231198 (PMC7188301; doi:10.1371/journal.pone.0231198)
Supplement: S1 Table — (DOCX) [file pone.0231198.s005.docx]

Table S1 Variance for principal component analysis

|  | Df | ChiSquare | F | P |
| --- | --- | --- | --- | --- |
| Model | 10 | 0.3968 | 18.419 | <0.001*** |
| Residual | 16 | 0.0345 |  |  |
