# Supplementary material for: The responses of soil bacterial communities and enzyme activities to the edaphic properties of coal mining areas in Central China
Source: PLoS One. 2020 Apr 28;15(4):e0231198. doi: 10.1371/journal.pone.0231198 (PMC7188301; doi:10.1371/journal.pone.0231198)
Supplement: S2 Table — (DOCX) [file pone.0231198.s006.docx]

Table S2 Significant variance analysis of eigenvalues

|  | Df | ChiSquare | F | P |
| --- | --- | --- | --- | --- |
| CCA1 | 1 | 0.2905 | 164.905 | <0.001*** |
| CCA2 | 1 | 0.0527 | 29.927 | <0.001*** |
| CCA3 | 1 | 0.0459 | 26.042 | <0.001*** |
| CCA4 | 1 | 0.0051 | 2.901 | 0.769 |
| CCA5 | 1 | 0.0016 | 0.960 | 0.997 |
| CCA6 | 1 | 0.0002 | 0.085 | 1.000 |
| Residual | 20 | 0.0352 |  |  |
