# Supplementary material for: The responses of soil bacterial communities and enzyme activities to the edaphic properties of coal mining areas in Central China
Source: PLoS One. 2020 Apr 28;15(4):e0231198. doi: 10.1371/journal.pone.0231198 (PMC7188301; doi:10.1371/journal.pone.0231198)
Supplement: S3 Table — (DOCX) [file pone.0231198.s007.docx]

Table S3 Coefficient variance analysis of principal components of environmental factors

|  | Df | ChiSquare | F | P |
| --- | --- | --- | --- | --- |
| pH | 1 | 0.1170 | 54.313 | *** |
| AP | 1 | 0.1028 | 47.699 | *** |
| TP | 1 | 0.0359 | 16.654 | *** |
| OM | 1 | 0.0004 | 0.196 | 0.862 |
| TN | 1 | 0.0088 | 4.088 | 0.03* |
| CAT | 1 | 0.0260 | 12.087 | 0.001*** |
| US | 1 | 0.0963 | 44.721 | 0.001*** |
| AlP | 1 | 0.0008 | 0.360 | 0.742 |
| INS | 1 | 0.0005 | 2.330 | 0.105 |
| Residual | 16 | 0.0345 |  |  |
